# Restitution of gene expression and histone acetylation signatures altered by hepatitis B virus through antiviral microRNA-like molecules in nontransformed murine hepatocytes

**DOI:** 10.1186/1868-7083-6-26

**Published:** 2014-11-14

**Authors:** Andreas CW Jenke, Kai O Hensel, Andreas Klein, Lisa Willuhn, Susanna Prax, Patrick P Weil, Theodor Winkler, Timo Deba, Valerie Orth, Armin Baiker, Stefan Wirth, Jan Postberg

**Affiliations:** Department of Paediatrics, HELIOS Medical Centre Wuppertal, Centre for Clinical and Translational Research (CCTR), Faculty of Health, Centre for Biomedical Education and Research (ZBAF), Witten/Herdecke University, Heusnerstr. 40, 42283 Wuppertal, Germany; Bavarian Health and Food Safety Authority, Veterinaerstrasse 2, D-85764 Oberschleißheim, Germany

**Keywords:** histone deacetylation, sirtuins, CpG signaling, non-coding RNA, HBV, HCC

## Abstract

**Background:**

Virus-host interactions result in altered gene expression profiles in host cell nuclei and enable virus particle production, thus obligatorily involving changes in their epigenomes. Neither such epigenome changes nor their response to antiviral treatment have been extensively studied to date, although viral infections are known to contribute to the long-term development of severe secondary diseases, for example, hepatocellular carcinoma. This may be causally linked to virus-induced persistent plastic chromatin deformations.

**Results:**

We studied whether impaired hepatitis B virus (HBV) replication can lead to the restitution of epigenome signatures hypothesizing that hepatocytes alternatively could adopt a ‘memory’ state of the infection; that is, the chromatin could persist in a HBV-induced configuration potentially inheritable between dividing hepatocytes. We therefore determined epigenomic signatures and gene expression changes altered by HBV and the effects of suppressed HBV replication in nontransformed hepatocytes of newborn mice. Further we investigated differential histone acetyltransferase and histone deacetylase activities in HBV-negative and HBVpositive hepatocytes, as well as the effects of HBV suppression on gene expression and the chromatin landscape. We show that the expression of several genes and the chromatin landscape become altered upon HBV infection, including global hypoacetylation of H2A.Z and H3K9. Reporter assays monitoring the activities of histone acetyltransferases or histone deacetylases, respectively, suggest that hypoacetylation most probably depends on elevated sirtuin deacetylase activity, but not on class I/II histone deacetylases. Using *Micrococcus* nuclease to study the chromatin accessibility in met murine-D3 and hepatitis B virus met murine hepatocytes, we demonstrate that the observed differences in H2A.Z/H3K9 acetylation lead to global chromatin structure changes. At all selected sites examined by chromatin immunoprecipitation and quantitative real-time PCR, these effects can be partly restituted via the nucleoside analog reverse transcriptase inhibitor 3TC or using anti-HBV microRNA-like molecules.

**Conclusions:**

Increased sirtuin activity might lead to global histone hypoacetylation signatures, which could contribute to the HBV-induced pathomechanism in nontransformed hepatocytes. Using several techniques to suppress HBV replication, we observed restituted gene expression and chromatin signature patterns reminiscent of noninfected hepatocytes. Importantly, ectopic expression of antiviral short-hairpin RNA, but not microRNA-like molecules, provoked intolerable off-target effects on the gene expression level.

**Electronic supplementary material:**

The online version of this article (doi:10.1186/1868-7083-6-26) contains supplementary material, which is available to authorized users.

## Background

To enhance virus particle production, virus-host interactions lead to manipulation of gene expression patterns in the infected cells, which necessarily involve changes in the epigenome. Neither such virus-induced host epigenome changes nor the response of these changes to therapies have extensively been studied to date, although it seems obvious that over the long term, several viral infections can contribute to the development of severe secondary diseases, which may be causally linked with persistent plastic chromatin deformations. For example, enforcement of hepatitis B virus (HBV) replication over long periods can eventually contribute to the development of chronic hepatitis B, liver cirrhosis and hepatocellular carcinoma (HCC). Globally, more than 350 million people are chronically infected with HBV; thus chronic hepatitis B belongs to the list of most common infectious diseases [1]. The most common mode of HBV transmission is vertically from mother to child with up to 90% of the neonates born to hepatitis B surface antigen (HBsAg)-positive mothers running the risk of becoming infected [2]. Chronic disease occurs mainly when the infection happens early in life during childhood. Adults become chronically diseased in only 5 to 10% of cases, whereas chronification rates can be higher than 90% when infants become infected in the first six months of life. Possible sequels of chronic hepatitis B are liver cirrhosis and cancer. In fact, chronic hepatitis B correlates with a 37-fold elevated probability of developing HCC [3]. However, the molecular reasons for these age-related differences in chronification remain unknown to date. It is also not known whether antiviral interventions lead to a restitution of epigenome patterns in host cells manipulated by HBV. Alternatively, one could hypothesize that hepatocyte nuclei adopt a ‘memory’ state of the HBV infection even if viral replication is suppressed; that is, the chromatin might persist in an HBV-manipulated configuration, which could be inheritable between dividing hepatocytes and could contribute to elevated secondary disease susceptibility.

Apart from the outstanding clinical relevance of elucidating the early pathomechanisms of HBV infection, this pathogen can serve as minimalistic model for virus-host interactions in general. The small 3.0 to 3.2 kbp HBV genome encodes only four genes, giving rise to seven proteins: *HBVgp1* (polymerase), *HBVgp2* (large S protein/middle S protein/S protein), *HBVgp3* (X protein) and *HBVgp4* (precore/core protein). The genome consists of partially double-stranded DNA, which is replicated via an RNA intermediate by reverse transcription through the HBV polymerase. Chronic HBV infections are accompanied by the persistence of the viral genomic covalently closed circular DNA (cccDNA) as stable episomes within hepatocyte nuclei, serving as a template for viral protein expression [4]. It is assumed that the X protein (HBx) acts as a transregulator and contributes to the malignant transformation of hepatocytes, and evidence increases that hepatocyte transregulation involves chromatin-modifying mechanisms, which act on the epigenomes of both the cccDNA and the host cells. HBx enrichment apparently leads to elevated DNA methyltransferase (DNMTs) levels [4–6]. In hepatocellular carcinoma cells transfected with wild-type HBV genomes, the histone acetyltransferase (HAT) p300 becomes recruited to the cccDNA. Concomitantly, levels of acetylated histones H3 and H4 (H3ac/H4ac) associated with the cccDNA increase and viral replication is enhanced. In contrast, p300-binding and H3ac/H4ac are reduced in hepatoma cells expressing a nonfunctional HBx [5]. Moreover histone deacetylases (HDACs) become recruited to the cccDNA via HBx, suggesting that this protein is critical for chromatin structure regulation of the cccDNA [4].

Although a few available studies underline the relevance of chromatin-modifying mechanisms for episomal stability of the cccDNA, HBV replication, viral gene expression and host cell reprogramming, most of that data resulted from studies in HCC cell lines or biopsies. In those samples, early epigenetic trans-acting decisions are indistinguishable from later events, and most models do not focus on the increased chronification susceptibility in infected infants. As a consequence, spatiotemporal dynamics of regulatory networks at the onset of chronic hepatitis B infection are not yet understood, but might be relevant for therapy decisions and antiviral drug development. To contribute, we compared the abundance of 80 mRNAs corresponding to genes relevant for HCC development in HBV-negative and HBV-positive nontransformed hepatocytes, which derived from newborn mice. We analyzed underlying chromatin signatures and investigated the response of hepatocytes upon impaired HBV replication using nucleoside analogs or RNA interference.

## Results

### Accumulation patterns of several messenger RNAs differ between noncancer and hepatocellular carcinoma hepatocytes upon chronic hepatitis B virus infection

It is clear that the time-displaced development of hepatocellular carcinoma can be an endpoint of chronic hepatitis B infection, and numerous piloting studies in the field have already compared gene expression profile differences in cell line models such as HepG2 hepatoma cells and HBV-positive HepG2.2.15 cells. However, the differences between chronic nontransformed HBV-positive hepatocytes and transformed HBV-positive hepatocytes have not been extensively studied so far. To confirm the importance of these differences, we studied the relative differential accumulation of numerous messenger RNAs (mRNAs) in HBV-positive murine liver biopsies (1.2.32 (Tg [HBV 1.3 genome] Chi32) lineage), which corresponded to more than 80 genes associated with liver cancer development and housekeeping genes by quantitative real-time polymerase chain reaction (qPCR) (Table 1; [see Additional file 1: Figure S1A]). The biopsies were obtained from 24-month-old mice (n = 5), which did not develop HCC or which developed HCC (N = 5). Our analyses showed - depending on whether *HSP90AB1* and *ACTB* were used for normalization or, alternatively, a combination of *HSP90AB1*, *ACTB* and *GAPDH* was used - that approximately 11 to 24% of the examined mRNAs were differentially deregulated. This observation highlights why it is reasonable to focus on nontransformed models of HBV infection in order to elucidate mechanisms in the hepatocellular regulome, which become modulated by HBV and potentially contribute to the development of HCC.

**Table 1:** **Overview of experimental and literature evidence for several differentially expressed genes and CpG signaling in mouse and humans**

	Changes in gene expression			Changes in CpG signaling		
Gene/function	HBV-Met versus MMH-D3	human chronic hepatitis B	human HCC	HBV-Met vs. MMH-D3	human hepatitis B/HCC	References
*CDH13* Regulation of cell-cell contacts	up (*P* <0.01)	no Pubmed hit; upregulation observed in chronic HBV infection (n = 3) versus HBV-negative liver biopsies (n = 3), *P* <0.05	no Pubmed hit	low 5meC high 5hmeC	hyper5meC in tumor (HCC)^1^	1 Yu, BMC Cancer. 2002 PMID: 12433278
*KDR (VEGFR2)* Regulation of angiogenesis	uncertain (up, *P* >0.05)	no Pubmed hit; no significant change observed in chronic HBV infection (n = 3) versus HBV-negative liver biopsies (n = 3)	up^2,3,4^	Na	no Pubmed hit	2 Yoshiji, Hepatology. 2001 PMID: 11283848 (**mouse**) 3 Shimamura, J Gastroenterol Hepatol. 2000 PMID: 10921418 4 Yoshiji, Hepatology. 1999 PMID: 10534339
*IGFBP1* IGF regulation/proliferation	uncertain (up, *P* >0.05)	no Pubmed hit; no significant change observed in chronic HBV infection (n = 3) versus HBV-negative liver biopsies (n = 3)	no Pubmed hit	Na	no Pubmed hit	Na
*IGFBP3* IGF regulation/proliferation	uncertain (up, *P* >0.05)	no Pubmed hit; no significant change observed in chronic HBV infection (n = 3) versus HBV-negative liver biopsies (n = 3)	normal^5^	Na	no Pubmed hit	5 Adamek, Oncol Rep. 2013 PMID: 23784592
*CDKN2A* Tumor suppressor	down (*P* = 0.038)	down^7,8^	down^6,7,8^	low 5meC moderate 5hmeC	hyper5meC (hepatitis B + HCC)^1,6,7,8^	1 Yu, BMC Cancer. 2002 PMID: 12433278 6 Li, Clin Cancer Res. 2004 PMID: 15569978 7 Kaneto, Gut 2001 PMID: 11171828 8 Shim, Cancer Lett 2003 PMID: 12565176
*WT1* Tumor suppressor	down (*P* <0.01)	down early: normal hepatocytes expressing HBx^5^/HBV-positive biopsies^5^ **versus** ‘significant upregulation observed in chronic HBV infection (n = 3) versus HBV-negative liver biopsies (n = 3)’	up^9,10,11^	Na	hyper5meC (HCC)^12,13^	5 Wu, Oncogene. 2001 PMID: 11439330 9 Uesugi, J Gastroenterol. 2013 PMID: 23142971 10 Perugorria, Cancer Res. 2009 PMID: 19190340 11 Sera, Eur J Cancer 2008 PMID: 18255279 12 Yu, Cell Res. 2003 PMID: 14672555 13 Zhang, Clin Cancer Res. 2007 PMID: 17289889
*DLC1* Tumor suppressor	down (*P* <0.01)	significant upregulation observed in chronic HBV infection	down^14,15,16^	Na	elevated 5meC (HCC)^15^	14 Dong, Cancer Epidemiol. 2009 PMID: 19766077 15 Wong, Cancer Res. 2003 PMID: 14633684 16 Leung-Kuen, PLoS One 2013 PMID: 23826380

*Abbreviations*: *CDH13* cadherin 13, *CDKN2A* cyclin-dependent kinase inhibitor 2A, *DLC1* deleted in liver cancer 1 IGF, insulin-like growth factor, *IGFBP1/3* insulin-like growth factor binding protein 1/3, *KDR* kinase insert domain receptor, *VEGFR2* vascular endothelial growth factor receptor 2, *WT1* Wilms tumor 1.

### Selective gene deregulation events are associated with chronic hepatitis B virus infection in nontransformed hepatocytes

**Figure 1:**
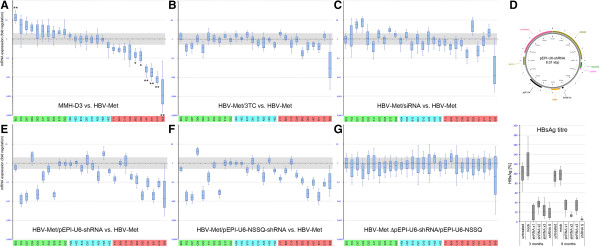
**Gene expression analyses in hepatitis B virus met murine hepatocytes** **(HBV-Met) with suppressed hepatitis B virus (HBV) replication versus untreated HBV-Met. A**. Relative enrichment of mRNA in MMH-D3 versus HBV-Met. H02 to H05 were used for normalization. **B**. 3TC-treated HBV-Met versus HBV-Met. **C**. HBV-Met treated with siRNA versus HBV-Met. **D**. pEPI-U6-shRNA and assessment of HBsAg. **E**. HBV-Met treated with antiviral short-hairpin RNA (shRNA) versus HBV-Met. **F**. HBV-Met treated with nonesense shRNA versus HBV-Met. **G**. Signal subtraction results from D and E. **A**-**G**. Statistical data are represented as boxplots displaying median fold-differences, interquartile range, and minimum/maximum values. Gray-shaded: fold-change range between -2.0x/+2.0x. Green: Ten most upregulated genes; red: Ten most downregulated genes. Cyan: 8 selected stably expressed genes. Fold-changes >1 indicate upregulation in HBV-Met versus MMH-D3; fold-changes <1 indicate downregulation. **P ≤0.01; *0.01 ≤ *P* ≤0.05.

To study gene expression changes and underlying epigenome signatures in nontransformed hepatocytes upon chronic HBV infection, which might possibly be relevant for HCC development, we made use of the immortalized, nontransformed murine hepatocyte cell lines met murine hepatocytes (MMH)-D3 (HBV-negative) and hepatitis B virus met murine hepatocytes (HBV-Met) (HBV-positive), which were derived from newborn mice [7, 8]. We analyzed the expression of over 80 liver cancer-related genes using qPCR arrays on reverse-transcribed RNA isolated from five biological replicates of MMH-D3 and six biological replicates of HBV-Met cell lines. Whereas most mRNAs did not exhibit significant differences in their relative abundance, *CDH13* (B03) was significantly up-regulated in HBV-Met. In contrast, *CDKN2A* (B06), *WT1* (G10) and *DLC1* (B11) were downregulated in HBV-Met when compared to MMH-D3 (Table 1; Figure 1A; [see Additional file 1: Figure S1B]) (all *P* <0.05). Several other mRNAs showed a tendency to be up- or downregulated, whereas most of these fold-changes did not reach significance (*P* ≥0.05). *KDR* (D07), *IGFBP1* (D03) and *IGFBP3* (D04) seemed to be slightly upregulated. In contrast, *IGF2* (D02), *CDH1* (B02), *fragile histidine triad protein*/*FHIT* (C06), *gluthatione S-transferase pi 1*/*GSTP1* (C10), *cyclin-dependent kinase inhibitor 1A*/*CDKN1A* (B04) and *signal transducer and activator of transcription 3*/*STAT3* (F11) seemed to be slightly downregulated. Also *beta-glucuronidase*/*GUSB* (H01), which was referred to as the housekeeping gene in the array, was significantly downregulated. We therefore decided not to use *GUSB* for normalization. Remarkably, we recognized that more genes became downregulated than upregulated. We were then interested in whether our observations made on the MMH-D3 and HBV-Met cells also hold for infected humans. To evaluate, we compared the expression of selected human genes between three HBV-negative and three HBV-positive age- and gender-matched adolescents (non-HCC) [see Additional file 1: Figure S1C] and gathered information from the literature (Table 1). The HBV-negative control group exhibited elevated transaminases, justifying diagnostic needle aspiration liver biopsies. Eventually, no evidence for liver diseases was found in any of the cases. In agreement with our observations in the MMH-D3/HBV-Met mouse cell system, *CDH13* mRNA was significantly enriched in HBV-positive specimens, but *IGFBP3* also seemed to be slightly upregulated. Concomitantly, *DLC1* and *CDKN2A* mRNAs were significantly downregulated. No significant difference was found for the accumulation of *IGFBP1* and *KDR* mRNAs between the HBV-positive and HBV-negative juvenile liver samples, whereas *WT1* was highly enriched in the HBV-positive specimens. Remarkably, this was the only observed stark contrast between cultivated nontransformed murine hepatocytes and human samples.

### Antiviral treatment entails partial restitution of met murine hepatocytes-D3-like expression patterns, but also causes severe side effects when short-hairpin RNA is used for hepatitis B virus suppression

To verify the functional connection between the presence of the HBV genome and the observed differences in gene expression patterns, we suppressed HBV replication in HBV-Met using different approaches. Thereafter, we compared the expression of selected genes in these cells with untreated HBV-Met (Figure 1A,B,C,E-G). With respect to our initial experiment, these genes were selected from three groups. Group 1 was composed of the ten most upregulated genes in the HBV-Met, group 2 of the ten most downregulated genes, and group 3 was composed of eight stably expressed genes, including four housekeeping genes.

**Figure 2:**
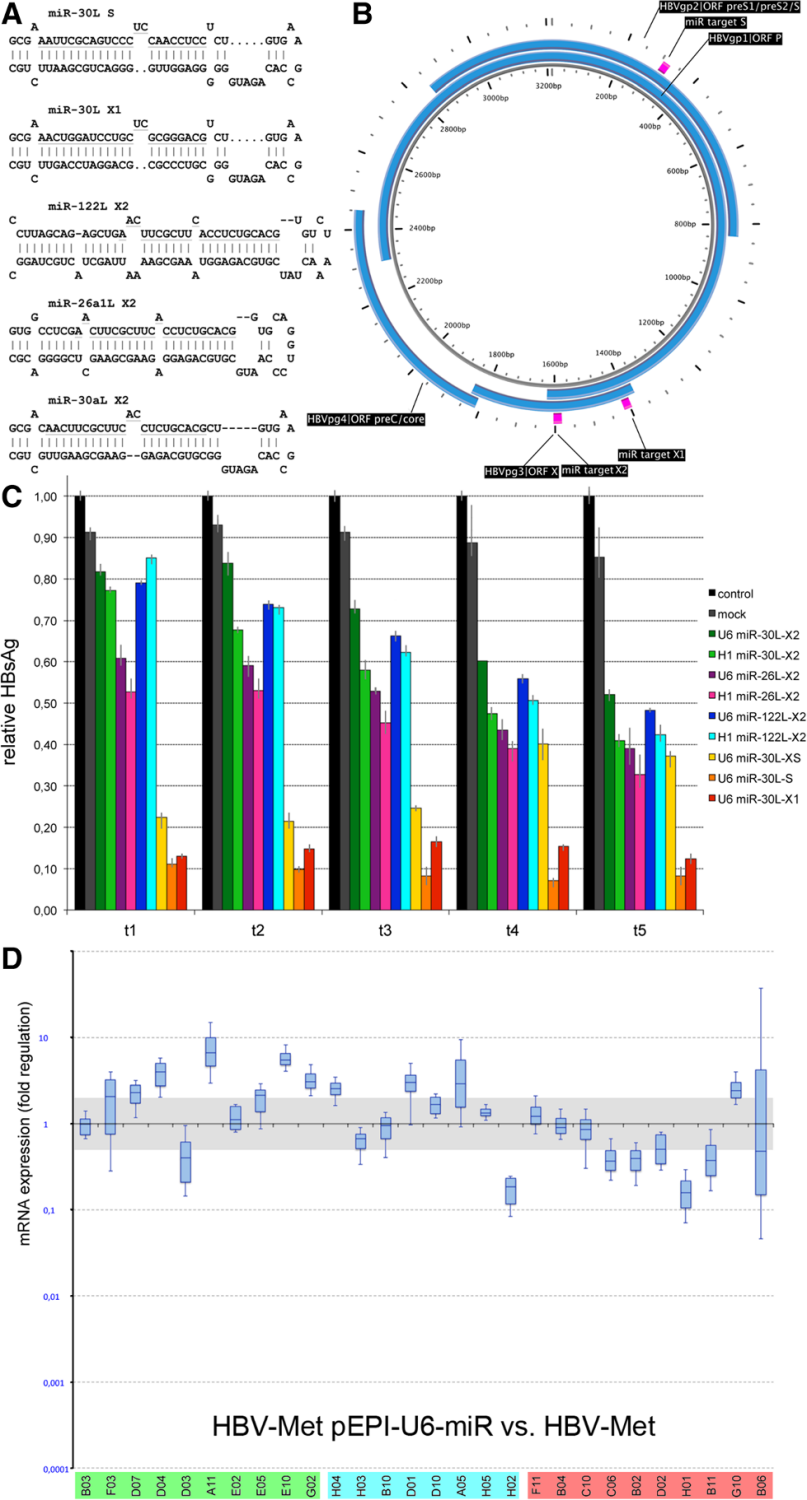
**Suppression of hepatitis B virus (HBV) replication using microRNA (miRNA)-like molecules. A**. Stem-loop structures of hsa-miRs containing antiviral target sequences. **B**. HBV transcripts and corresponding target sequences on the covalently closed circular DNA (cccDNA). **C**. Hepatitis B surface antigen (HBsAg) assessment upon hepatitis B virus met murine hepatocytes (HBV-Met) treatment with candidate miRs. **D**. Gene expression analyses in HBV-Met treated with miRNA-30 L-X1 versus untreated HBV-Met. Statistics and coloring correspond to Figure 1A-G.

Firstly, we treated HBV-Met with the nucleoside analog reverse transcriptase inhibitor 2′,3′-dideoxy-3′-thiacytidine (3TC/lamivudine) (Figure 1B), which is widely used for chronic hepatitis B treatment. Whereas expression patterns of most genes examined did not differ significantly from HBV-Met, three of the genes upregulated in HBV-Met versus MMH-D3 those that were downregulated in the 3TC-treated HBV-Met (*reelin*/*RELN*/F03, *KDR*/D07, and *IGFBP1*/D03). Remarkably, *CDKN2A* (B06) and *CDH1* (B02) exhibited differential expression patterns reminiscent of HBV-Met when compared with the HBV-negative MMH-D3, whereby both genes were downregulated or silenced in HBV-Met (Figure 1B). To complement this experiment, we utilized RNA interference to target HBV transcripts in HBV-Met, which in theory enables more specific suppression of HBV replication. Initially we selected putative siRNA candidate sequences against sites encoded within ORF P, ORF S or ORF X and a mock small interfering RNA (siRNA) without homology to human or HBV transcripts (nonsense siRNA). These siRNAs were transfected into HBV-Met prior to periodical quantification of HBsAg in cell culture supernatants over 5 days. In HBV-Met cells the most efficient siRNAs against ORF X transcripts tested led to an approximately 80% reduction of HBV replication as deduced by HBsAg measurements in the supernatant. We purified RNA from these cells to perform qPCR arrays analyses as described. Expression patterns of several genes examined were reminiscent of those obtained, when HBV-Met were compared with MMH-D3 (Figure 1C). In detail *KDR* (D07), *IGFBP3* (D04) and *protein tyrosine kinase 2*/*PTK2* (E10) were upregulated after siRNA treatment, whereas *CDH1* (B02), *GUSB* (H01) and *CDKN2A* (B06) were downregulated. All group 3 genes remained stable. To study longer term effects, we performed experiments using antiviral small RNA molecules expressed from optimized pEPI-RNAi derivatives [9, 10]. We transfected different pEPI-U6-small hairpin RNA (shRNA) constructs into HBV-Met. HBV replication in HBV-Met was monitored after growth for 3 and 8 months, respectively, showing that HBV suppression was efficient and stable (Figure 1D). Subsequently we analyzed gene expression in HBV-Met 3 months after transfection with pEPI-U6-shRNA-X1. Upon expression of the antiviral shRNA dramatic expression changes for many of the genes examined were observed (Figure 1E-G). Over 50% of the examined genes appeared to be downregulated in shRNA-treated HBV-Met, when compared to untreated controls. These patterns differed starkly from MMH-D3. To test whether the observed effects were target sequence-specific, a nonesense shRNA expressing pEPI vector (Figure 1F) or solely the pEPI-luciferase backbone was established in HBV-Met cells [see Additional file 1: Figure S2]. These controls revealed that the pEPI-luciferase vector alone caused at best minor off-target effects, whereas interestingly, the expression of nonsense-shRNA caused almost congruent gene expression patterns as described for the use of HBV-directed shRNAs (Figure 1E, F). Strikingly, after the subtraction of fold-changes caused by nonsense-shRNA from fold-changes caused by HBV-directed shRNA, none of the genes investigated showed significant differences, suggesting that almost all observed gene expression changes were directly and specifically induced by shRNA (Figure 1G). Only *CDKN2A* (B06) expression appeared to be diminished in HBV-Met when compared to shRNA-treated HBV-Met. These off-target effects obviously induced by shRNA expression clearly reduced the value of such experiments for the study of HBV-induced changes in the transcriptome of host cells and for clinical application. We therefore decided to camouflage the antiviral target sequences as the cell’s own microRNA (miRNA) in similar ways, as miRNA-30-like precursors have been used for the study of gene function before [11] to circumvent a hypothetical cellular response mechanism. With respect to miRBASE [12], we designed stem-loop hsa-miRNA-like oligonucleotides and cloned them into the pEPI vector system, giving rise to pEPI-U6-miR/pEPI-H1- miRNA plasmids (Figure 2A, B).

To optimize the use of antiviral miR, we constructed alternative pEPI-miRNA vectors using different miRNA templates, which were reportedly expressed in human liver (Figure 2A) [12], in combination with either the human H1 or U6 RNA polymerase III promoter. Furthermore we made use of different antiviral target sequences to replace mature 5p-miRNA sequences within the stem-loop precursors (Figure 2B). After transfection into HBV-Met of HBsAg quantities in the cell culture supernatant were assessed over a period of 5 days (Figure 2C). We noted that HBsAg concentrations were slightly lower using the H1 promoter construct. Also HBsAg suppression was slightly more efficient, when a miRNA-26-like construct was used, whereby miRNA-122-like and miRNA-30-like constructs exhibited similar efficiency. Target sequence selection was apparently more relevant for the HBV suppression potency of miRs (target X1 > X2 > S). Eventually we selected a pEPI-U6-miRNA-30-like clone targeting transcripts of HBV ORF X/ORF P for further experiments. Following transfection into HBV-Met, selection and growth over 3 months, we performed comparative gene expression studies (Figure 2D). Strikingly, the pronounced off-target effects on hepatocyte gene expression patterns seen in the shRNA-treated HBV-Met were not observed after episomal miRNA expression. Moreover, gene expression patterns of several genes were reminiscent of the patterns obtained when HBV-negative MMH-D3 were compared with HBV-positive HBV-Met.

### Reversible hepatitis B virus-induced hypoacetylation shapes the epigenomic landscape in nontransformed hepatocytes

**Figure 3:**
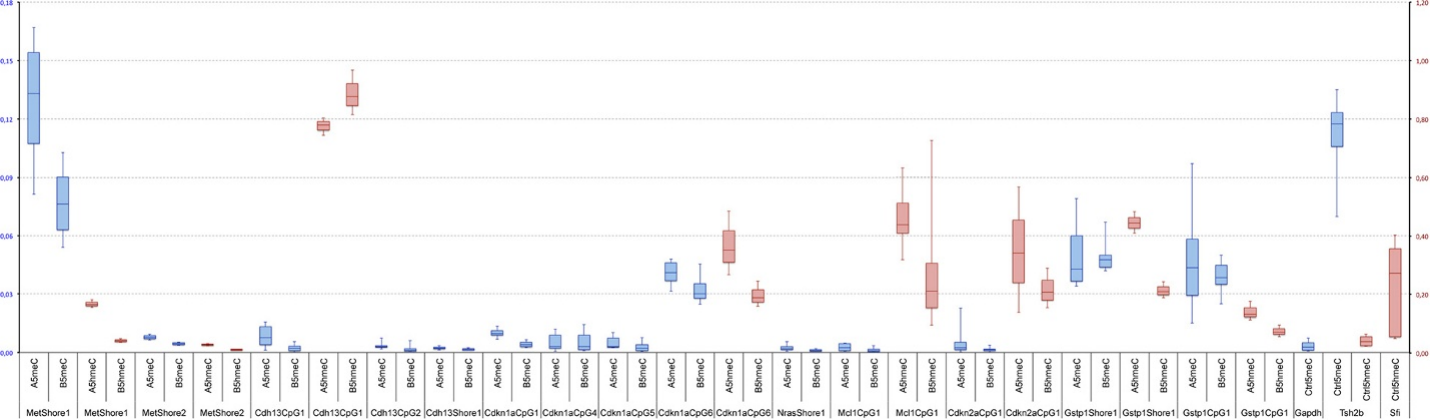
**Recovery of 5meC and 5hmeC at selected sites.** MMH-D3 (‘A’ as part of the x-axis caption) versus hepatitis B virus met murine hepatocytes (HBV-Met) (‘B’ as part of the x-axis caption). Blue boxes: 5meC; red boxes: 5hmeC. Data are presented as median fold-differences, interquartile range, and minimum/maximum values.

**Figure 4:**
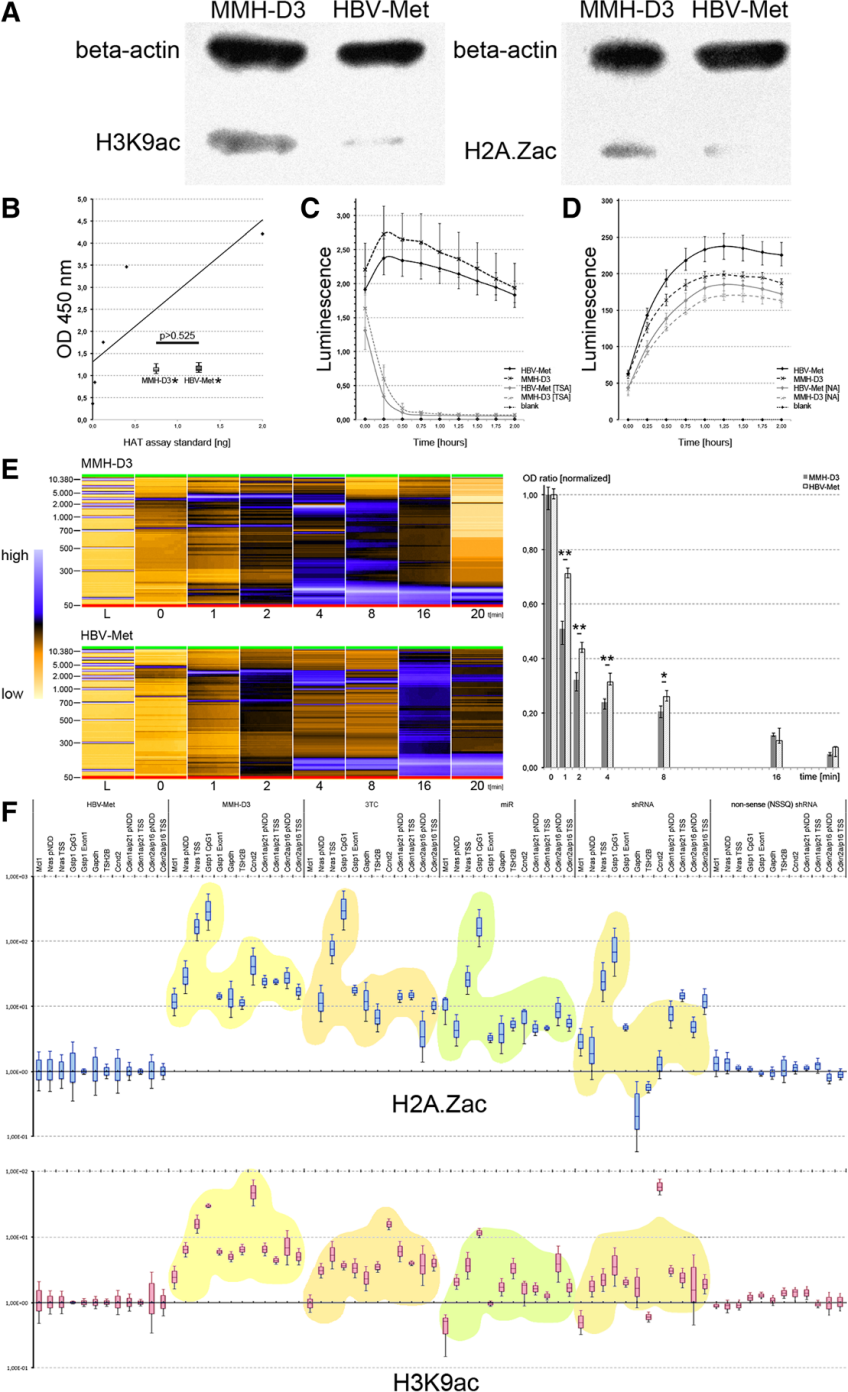
**Comparative analyses of histone acetylation and chromatin structure in met murine hepatocytes** (**MMH)-D3 versus hepatitis B virus met murine hepatocytes (HBV-Met) cells. A**. Western blot analyses of H3K9ac (left) or H2A.Zac (right) enrichment in MMH-D3 and HBV-Met cells. Beta-actin was used as loading control. **B**-**D**. Assays of histone acetyltransferase (HAT)/ histone deacetylase (HDAC) activities. **B**. HAT activity ELISA using nuclear extracts from 10.000 of each of both cell lines MMH-D3 and HBV-Met. The dots and the trend line correspond to the kit’s standards, whereas the boxplots (referring to the y-axis only) represent the results of several measurements (n =10 for each cell line) using MMH-D3 or HBV-Met cells. **C**. Results of class I/II HDAC activity measurements over time using 10.000 cells from each of the two cell lines for luminescence reporter assays. **D**. Results of class III HDAC activity (sirtuins) measurements over time using 20.000 cells from each of the two cell lines for luminescence reporter assays. **E**. Results of MNase digests over time using isolated nuclei from 1x 10^6^ cells per time point. **F**. Fold-change differences of H2A.Zac/H3K9ac recovery at selected sites. HBV-Met versus MMH-D3 or HBV-Met with suppressed hepatitis B virus (HBV) replication. For each site recovery from HBV-Met was used for normalization.

Since chromatin, rather than naked DNA, is the substrate for gene expression in the cell nucleus, the ability of viruses to direct chromatin modifications is a prerequisite for reprogramming the host cell transcription machinery for virus particle production [13]. In this context, it is notable that acquired epigenetic abnormalities possess similar relevance for malignant transformation of cells to mutations in the genome [14]. Thus changes in the epigenomic signature implemented by chronic viral infections might harbor the risk of malignant transformation. We therefore investigated whether epigenomic markers associated with several sites within promoters of selected genes, such as DNA cytosine methylation (5meC) or post-translational modifications of histones (PTM), became differentially manipulated by HBV. Using methylated DNA immunoprecipitation (MeDIP) in combination with qPCR, we analyzed whether selected CpG-rich ‘island’ or ‘shore’ sites localized within the promoters of several genes could be pulled-down using 5meC specific antibodies (Figure 3). These putative DNA methylation target sites were associated with selected genes differentially repressed in HBV-Met (for example, *CDKN2A*, *CDKN1A*) or with genes being previously described as hypermethylated in HCC (for example, *GSTP1* and *CDH13*) [15, 16]. We were surprised to some extent that we did not observe obvious differences of 5meC enrichment, whereby most sites were either hypomethylated or exhibited an intermediate level of DNA methylation in both cell lines. As expected, the control genes used were detected being either hypomethylated (*glyceraldehyde-3-phosphate dehydrogenase*/*GAPDH*) or hypermethylated (*testis-specific histone 2B*/*TSH2B*). We speculated that previously reported DNA hypermethylation could be a false-positive artifact, which could result from the enrichment of hydroxymethylated cytosines at sites being investigated. Bisulfite-dependent methods are known to be prone to false-positive detection of methylated cytosines in cases where the putative DNA demethylation intermediate 5-hydroxymethylcytosine (5hmeC) is highly enriched [17]. To complement and to test whether relevant 5hmeC levels were detectable, we applied 5hmeC-specific antibodies for immunoprecipitation. Again, no obvious differences were seen in HBV-Met versus MMH-D3 (Figure 3). With respect to a hydroxymethylated control gene (*splicing factor 1*/*SF1*), we detected biologically relevant enrichment of 5hmeC at several sites in the promoters of *CDKN2A*, *CDKN1A*, *GSTP1*, *CDH13* and *myeloid cell leukemia sequence 1*/*MCL1*. Since no differential patterns responsible for the transregulation of selected genes by HBV were seen on the DNA methylation/hydroxymethylation levels, we went on to study putatively differential patterns of histone modifications (Figure 4). Earlier studies reported a linkage between HBV infection and HAT (p300) or HDAC recruitment in hepatocytes. As an initial approach we thus selected H2A.Zac as well as H3K9ac as markers to study possible epigenome-wide differences in nuclear extracts from HBV-Met and MMH-D3 cells by Western blot analyses (Figure 4A). Interestingly, we found that both H2A.Zac and H3K9ac were enriched in MMH-D3 cells, when compared to HBV-Met cells. We assumed that histone hypoacetylation could theoretically result from either reduced histone acetyltransferase activity or from elevated histone deacetylase activity or from a combination of all. We therefore performed various reporter assays to get insight into differences in relevant chromatin modifying activities in HBV-Met cells versus MMH-D3 cells. We could not observe significant differences in the activities of HATs (Figure 4B) or class I and II HDACs (Figure 4C) between both hepatocyte lines. Interestingly, we detected significantly increased relative activity of the NAD^+^-dependent HDAC class III enzymes (sirtuins) (Figure 4D). We were next interested in whether one or several of the seven murine sirtuin genes present in the mouse genome were differentially regulated between HBV-Met and MMH-D3 cells. As a preliminary approach, we performed qPCR, measuring the differential accumulation of SIRT1-7 mRNAs in both cell lines. Notably we observed that the mRNAs of SIRT1, SIRT2, SIRT4 and SIRT7 were slightly but significantly (*0.01 ≤ *P* ≤ 0.05) elevated in HBV-Met cells when compared with MMH-D3 cells, whereby SIRT3 and SIRT4 could not be detected in either cell line [see Additional file 1: Figure S3]. In summary, both observations - the decreased presence of H2A.Zac and H3K9ac, as well as the elevated relative sirtuin deacetylase activity in HBV-Met hepatocytes - suggest that the observed hypoacetylation at specific sites reflects a global, epigenome-wide effect.

Since histone acetylation is generally believed to be associated with a decondensed chromatin structure, we hypothesized that - as a structural consequence of the increased sirtuin deacetylase activity in HBV-Met cells - global histone hypoacetylation could favor a condensed chromatin structure, leading to reduced chromatin accessibility in HBV-Met cells. Alternatively the observed histone hypoacetylation could also be explained by reduced nucleosome occupancy in the HBV-positive hepatocytes, which would be reminiscent of the age-related thinning of nucleosome occupancy in single-celled eukaryotes and mammals [18]. In theory, such reduced nucleosome occupancy could lead to enhanced accessibility of chromatin. We therefore assessed the chromatin accessibility over time using *Micrococcus* nuclease (MNase) assays. Using microcapillary electrophoresis, we observed that the enrichment of DNA bands corresponding to mono-, di- and trinucleosomes was deferred in HBV-Met when compared with MMH-D3 (Figure 4E, left). We further monitored the intensity ratios between the undigested gDNA and the DNA band, which corresponded to mononucleosomes (Figure 4E, right). These intensity ratios were significantly lower at time points 1, 2, 4 and 8 minutes, showing that chromatin purified from MMH-D3 cells was more accessible for MNase when compared to HBV-Met chromatin. These results provided strong evidence that the detected histone hypoacetylation led to changes in the global chromatin structure of HBV-Met cell nuclei.

In order to study the observed differences in the turnover of H2A.Zac and H3K9ac in more detail and to elucidate the influence of HBV-suppressive interventions, we went on to analyze histone PTM patterns by chromatin immunoprecipitation (ChIP) and qPCR (Figure 4F). Therefore we selected 12 sites within the promoter of eight genes as anchor points. Strikingly, at all sites examined, we discovered highly significant hypoacetylation of H2A.Z and H3K9 in HBV-Met versus MMH-D3. To analyze whether these acetylation pattern changes were due to the presence or replication activity of HBV, we made use of several suppression techniques as described above. Importantly, upon impaired HBV replication, these experiments showed that chromatin signatures reminiscent of HBV-negative MMH-D3 were apparently restituted with varying quality for both H2A.Zac and H3K9ac. The best apparent quality was particularly achieved when the anti-HBV miRNA-like molecules mimicking hsa-miR30a were used. In contrast, the expression of a nonesense miRNA did not lead to changed H2A.Zac and H3K9ac patterns that were distinguishable from untreated HBV-Met.

## Discussion

To study the mechanisms relevant for chronification of hepatitis B with an emphasis on infantile HBV infections and the possible later malignant transformation of hepatocytes in the course of chronic HBV infection, it is reasonable to utilize experimental nontransformed models instead of hepatoblastoma cells, which already exhibit carcinogenic modified profiles of their epigenomes and transcriptomes. We therefore used the primary hepatocyte lines HBV-Met and MMH-D3. The latter derived from primary hepatocytes of newborn transgenic mice expressing the *hepatocyte growth factor receptor*/*MET* proto-oncogene, which encodes the transmembrane receptor, whose extracellular domain was truncated. This gave rise to apoptosis-resistant, immortalized but nontransformed, well-differentiated hepatocytes [8]. Immortalized primary HBV-Met hepatocytes were cultured from the newborn offspring of cyto-Met mice parental for MMH-D3, which were bred with HBV-transgenic mice. These mice replicated HBV from an integrated transcriptional template encoding all full-length HBV transcripts at a replication level comparable to patients with chronic hepatitis B [7]. These characteristics of both related hepatocyte lines made them a preferable *in vitro* model for the intended studies.

Focusing on genes potentially relevant for HCC development we detected upregulation of *CDH13*, *KDR*, *IGFBP1/3* and downregulation of *CDKN2A*, *WT1*, *DLC1* suggesting that transregulation of these genes might play a role in the early phase of malignant hepatocyte transformation. Interestingly, *CDH13* was earlier observed being silenced in HCC, but overexpressed in intratumoral endothelial cells. It was also noted that its overexpression could stimulate proliferation and motility of endothelial cells and was associated with *vascular endothelial growth factor*/VEGF-driven angiogenesis [19]. One VEGF-responsive receptor is encoded by *KDR*. Elevated expression of *KDR* was earlier assumed to be involved in the regulation of VEGF-mediated HCC development and angiogenesis at early and later stages of tumor development [20]. Our observation that *CDH13,* and possibly to some extent *KDR,* was among the most prominently upregulated genes in HBV-Met strengthens the hypothesis that VEGF-signaling in addition to other growth factor-mediated pathways is important in HBV-induced HCC development [21]. At the same time elevated *IGFBP3/1* levels as observed in our study possibly indicate a continuance of a competing growth-limiting response at this precancer stage. Apparently this response eventually diminishes, since others consistently reported it to be repressed in HCC [22, 23]. Concomitantly, tumor suppressor genes such as *CDKN2A*, *WT1* and *DLC1* became downregulated in HBV-Met. Shutdown of these genes was earlier associated with HCC or with tumors of other entities [24–26], but our findings highlight that targeted repression of these anti-proliferative genes might be tightly entangled with an early onset of malignancy. Notably, while we obtained very similar mRNA accumulation profiles in cultured murine hepatocytes and human liver samples for almost all differentially regulated genes, the observed deprivation of *WT1* mRNA in HBV-Met cells was in stark contrast to its significant enrichment in HBV-positive human liver samples. This accumulation in human non-HCC samples was reminiscent of several reported cases, where *WT1* mRNA was enriched in hepatocellular cell carcinoma [27–29]. In summary, a common set of genes seems to be deregulated in several HBV-positive cell models including HBV-Met, but some remarkable differences remain. This could at least partly be due to the chimeric nature of the HBV-Met model, which contains the human HBV genome integrated into a murine hepatocyte genome. Therefore, the relevance of several observations still has to be judged with care and will need further examination in future.

From a clinical perspective, however, it is desirable to know whether these changes are reversible upon HBV suppression. We therefore used several techniques to investigate whether knockdown of HBV replication could restore MMH-D3-like gene expression patterns. It seemed reasonable to make use of the nucleoside analog reverse transcriptase inhibitor 3TC because the HBV-Met cells replicate HBV from the integrated greater-than-genome-length HBV transcriptional template [7]. Upon 3TC treatment, most genes remained in a state reminiscent of chronic HBV infection, whereby the expression patterns of five genes downregulated in HBV-Met but none of the upregulated genes became restituted (Figure 1B). Targeted suppression of HBV by siRNA appeared more efficient, leading to the restitution of eight genes (each four out of 20 up- or downregulated) in HBV-Met (Figure 1C), whereby group 3 genes remained stable. However, since siRNA treatment allows only short-term observation, we went on to stably express antiviral RNA in HBV-Met. Earlier studies encouraged us to utilize shRNA against HBV, since this technique was applied before on HepG2.2.15 to study the suppressive potency of anti-HBV-shRNA, and the cytotoxic effects in this hepatoblastoma cell line reportedly appeared negligible [30]. In fact, we observed remarkable suppressive potency by means of HBsAg assessments in cell culture supernatants after 3 and 8 months during the observation period (Figure 1D). However, on the gene expression level, we observed massive deregulation effects with congruently deviating patterns using either an antiviral or a nonsense shRNA. These almost identical patterns and the omitted contribution of the shRNA-cassette-deficient vector on gene expression patterns suggested that these off-target effects were due to the shRNA in a sequence independent manner. In line with this, fatal off-target effects upon ectopic shRNA expression in mice livers were observed before and were attributed to the oversaturation of cellular RNA interference pathways [31]. It seems plausible that those observations and the reasons for off-target effects reported here could be mechanistically connected. Naturally, such pronounced off-target effects are intolerable for functional HBV-knockdown studies and certainly for future applications in chronic HBV therapies. To circumvent putative hepatocellular ‘friend or foe’ recognition, we mimicked hsa-miRNA-30-like molecules, which were compared to other miRNA-like constructs for their suppressive potency in prior experiments. Strikingly, using this miRNA-like construct expressed from the pEPI-U6-miRNA episome, we observed no off-target effects and a far better recovery of MMH-D3-like expression patterns. Specifically, eight out of ten genes upregulated in HBV-Met versus MMH-D3 were also upregulated in HBV-Met when compared with miRNA-treated HBV-Met, and five out of ten genes downregulated in HBV-Met versus MMH-D3 were also downregulated in HBV-Met versus miRNA-treated HBV-Met. These findings provide evidence for a functional connection between deregulation of these genes and the presence of HBV. We also hypothesized that the gene expression deregulation was a phenotypic consequence of epigenome plasticity modulated by HBV. Consequently, we analyzed HBV-induced promoter DNA methylation patterns in HBV-Met. To date, few studies focusing on limited numbers of selected genes are available for describing HBV-induced changes in the hepatocyte epigenome. Most of them were done in HCC biopsies or cell lines representing end stages of malignant transformation. Some data were available for *CDKN2A*, *GSTP1*, *CDKN1A* and some other genes [32–35], whereby these genes were reported to be downregulated by promoter CpG-island hypermethylation in HCC. Similarly, one study reported similar hypermethylation in the *CDKN2A* promoter in preneoplastic lesions. This led the authors to suggest that *CDKN2A* hypermethylation was an early event in HCC development [36]. Importantly, our data does not support this conclusion, as we could not detect DNA methylation pattern differences at any of the sites examined (Figure 3). Notably, we examined mouse homologous sites for *CDKN2A* and *GSTP1* that were mentioned in the cited literature. Moreover, we could not see any sign for hypermethylation in *CDKN2A*, whose DNA methylation level rather resembled that of hypomethylated *GAPDH*. Both sites examined in *GSTP1* reached intermediate DNA methylation levels if compared with a hypermethylated site in *TSH2B*. In contrast to all relevant studies cited utilizing techniques indiscriminative for DNA methylation or hydroxymethylation, we used specific antibodies against either 5meC or 5hmeC for immunoprecipitation. Interestingly, with respect to the hydroxymethylated control gene *SF1,* we observed 5hmeC enrichment at the same site in *CDKN2A*, which was hypomethylated. We discovered more instances for sites, where 5meC levels were low or moderately elevated, but at the same time 5hmeC was enriched (sites in *CDH13*, *CDKN1A*, *MCL1*, *CDKN2A*, *GSTP1*). This might explain some of the discrepancies above described, since a lack of discriminative techniques for 5meC and 5hmeC in the previous studies might have led to biased quantification of DNA methylation [17]. Further, the few studies available mostly focused on already transformed hepatocytes. Taking this into account, earlier results and our own data might not be mutually exclusive, but rather represent different stages in the malignant transformation process with DNA hypermethylation not being an initial but instead a late event for HBV-induced gene repression (for example, *CDKN2A*). To seek mechanistic explanations for the observed differential gene regulation, it was reasonable to study the turnover of specific histone PTMs at selected gene promoters. Strikingly, we found that turnover of histone acetylation of the histone variant H2A.Z or at lysine 9 of H3 differed at all sites examined between MMH-D3 and HBV-Met (Figure 4) with significant hypoacetylation in HBV-Met. This would be in agreement with the hypothesis that HDACs are involved in the HBV-induced repression of selected genes [4].

In order to get insight into the mechanism of HBV-induced histone hypoacetylation we tested several hypotheses. We assumed that the detected hypoacetylation could be due to one or more of the following mechanisms: 1) reduced HAT activity; 2) elevated HDAC activity; or 3) desolation of epigenomic signatures, for example, through reduced nucleosome occupancy. To test this, we measured the activities of several enzymes involved in the regulation of histone acetylation, that is, histone acetyltransferases and histone deacetylases (class I/II HDACs and class III HDACs/sirtuins). We observed that only the activity of the NAD^+^-dependent sirtuins (*silent mating type information regulation 2 homolog*/SIRT) differed between the MMH-D3 and HBV-Met cells, suggesting that particularly these class III HDACs could be involved in the HBV-driven histone hypoacetylation, which appeared to be an early pre-HCC event in hepatitis B. Our results indicate slightly elevated mRNA levels for *SIRT1*, *SIRT2*, *SIRT5* and *SIRT7* in HBV-Met cells when compared to MMH-D3, although the biological relevance of this observation remains undisclosed. It seems likely - rather than deducing differential histone acetylation levels from changes in the expression of sirtuin genes - that the deregulation of sirtuins could occur on the protein level, which might lead to changes in their chromatin recruitment or turnover, respectively. It is interesting in this context that two very recent studies report a physical association between SIRT1 with the HBV cccDNA and that SIRT1 is apparently tightly involved in the upregulation of viral transcription [37, 38]. It may be carefully speculated that upregulation of SIRT1 by HBV could lead to globally changed acetylation signatures in the host cell epigenome. Moreover, we showed that global histone hypoacetylation in HBV-Met cells correlated with changes in the chromatin structure. In detail, HBV-Met chromatin was significantly less sensitive to MNase, suggesting a globally more condensed chromatin structure.

Interestingly, at all selected sites examined by ChIP-qPCR, histone deacetylation in the HBV-Met cells could be partly restituted via a nucleoside analog reverse transcriptase inhibitor or by anti-HBV miRNA-like molecules mimicking hsa-miRNA-30a, whereas ectopic expression of antiviral shRNA seemed to provoke intolerable off-target effects on the gene expression level. In the future, this has to be taken into account with respect to the open problem, whether therapies lead to the restitution of regular chromatin signatures, or whether hepatocytes persist in a deregulated epigenetic ‘memory’ state of HBV infection and thus still carrying the risk for later malignant transformation.

## Conclusions

Taken together, our experiments demonstrated that an increased activity of sirtuins, which might lead to global histone hypoacetylation signatures, could contribute to the HBV-induced pathomechanism in nontransformed hepatocytes during hepatitis B. In contrast to studies in hepatoma cells, we could not find evidence for the importance of DNA hypermethylation as an early event, the involvement of HAT or class I/II HDACs on an epigenome-wide level or a desolation of the nucleosome landscape in the HBV-Met chromatin.

Our ChIP experiments, moreover, suggested that impaired HBV-replication by either 3TC or antiviral miRNA expression, and to a lesser extent by shRNA, apparently resulted in a restitution of H2A.Zac and H3K9ac signatures at different grades and at all sites examined. These results strongly suggested that the observed changes in chromatin structure were mechanistically driven by HBV or its transcription, respectively. The resulting patterns were reminiscent of MMH-D3 with only single sites occasionally remaining in an HBV-Met-like state. The best recovery of MMH-D3-like gene expression without observable off-target effects was observed in HBV-Met treated with miRNA-like molecules. We therefore concluded that the observed effects on the chromatin signature in HBV expressing hepatocytes were indeed directly induced by the presence/replicative activity of HBV.

## Methods

### Cells and specimens

MMH-D3 and HBV-Met cells were cultivated as described elsewhere [7, 8]. In some experiments, cells were treated with 25 μM 3TC (Sigma Aldrich, St. Louis, MO, USA). Pediatric human specimens were obtained from patients who routinely underwent diagnostic needle aspiration liver biopsy. Written informed consent for research material was obtained from all legal guardians. All work has been conducted according to the principles expressed in the Declaration of Helsinki. Mice liver samples of the 1.2.32 (Tg [HBV 1.3 genome] Chi32) lineage (HCC negative and HCC positive) were kindly donated by Ulrike Protzer, Institute of Virology, TU Munich, Germany.

### Hepatitis B surface antigen measurements

Hepatitis B surface antigen (HBsAg) concentrations in cell culture supernatants were quantified using the Elecsys HBsAg II ELISA test (Roche Diagnostics, Rotkreuz, Risch, Switzerland).

### Gene expression analyses

Prior to gene expression analyses, RNA was isolated from at least three biological replicates for each experiment using Trizol reagent (Sigma Aldrich, St. Louis, MO, USA) according to the manufacturer’s recommendations. RNA integrity was checked using an Agilent Bioanalyzer 2100 with the Agilent RNA 6000 Nano Kit for microcapillary electrophoresis (Agilent Technologies, Santa Clara, CA, USA). Using the RT^2^ First Strand kit (Qiagen/SABiosciences, Hilden, Germany), cDNA templates were synthesized from 1 μg RNA. Gene expression was analyzed from these cDNAs using RT^2^ Profiler PCR arrays (PAMM-133R; Qiagen/SABiosciences, Hilden, Germany), which contained validated primers for 84 genes [see Additional file 1: Figure S4] relevant for liver cancer development, 5 housekeeping genes and quality control primers for estimation of reverse transcription efficiency and genomic DNA contamination on a Corbett Rotor-Gene 6000 qPCR device (Qiagen, Hilden, Germany). In some experiments, we measured the accumulation of SIRT1-7 mRNA using the primer pairs displayed in Table 2. The housekeeping genes *ACTB*, *GAPDH* and *RPL19* were used for normalization. For relative comparative quantification of gene expression fold changes we applied the ΔΔCt method [39] using at least three housekeeping genes for normalization.

**Table 2:** **Primer pairs used for the detection of murine SIRT1-7 mRNAs by quantitative real-time polymerase chain reaction** (**qPCR)**

Name	Forward primer	Reverse primer	Amplicon size
mmuSIRT1	Ggccgcggataggtccata	acaatctgccacagcgtcat	136 bp
mmuSIRT2	Gtgcaggaggctcaggatt	tgtagcgtgtcactccttcg	163 bp
mmuSIRT3	Cgctaaacttctcccgggtt	cctgtaacactagtcctcgcc	156 bp
mmuSIRT4	Aacccgactgtttagccgtt	ccgctcattcttattctgtctgg	190 bp
mmuSIRT5	Caccgacagattcaggtttca	agtgccctgctttagcactc	155 bp
mmuSIRT6	Cacaaaacatgaccgccagg	ctgcaccattgagatgcacg	191 bp
mmuSIRT7	Tctacaaccggtggcaggat	cctcctaggatagggggagc	160 bp

### RNA interference

For RNA interference we used annealed duplex siRNA oligos and HiPerFect transfection reagent (Qiagen, Hilden, Germany). Long-term suppression using shRNA/miRNA was achieved using the pEPI vector system [9] with the luciferase-S/MAR transcription unit under control of the human hepatocyte-specific alpha 1-antitrypsin promoter (A1AT). The shRNA/miRNA targeted against HBV was expressed under control of the human U6 or H1 RNA polymerase III promoters downstream of the S/MAR cassette. Plasmids were transfected into HBV-Met using Lipofectamine 2000 (Life Technologies, Carlsbad, CA, USA), followed by G418 selection for 10 days. Sampling was done at the time points indicated below.

### Analyses of CpG signaling

DNA methylation/hydroxymethylation was assessed via methylated DNA immunoprecipitation (MeDIP/hMeDIP) as described previously [40]. Briefly, genomic DNA was isolated by phenol:choroform:isoamylalcohol extraction, sheared by ultrasonic treatment prior to pull-down of 5meC or 5hmeC containing fragments using antibodies selective for either methylated or hydroxymethylated DNA (Diagenode, Liège, Belgium). Relative enrichment of precipitated sequences in MeDIP was assessed using qPCR.

### Western blots

For Western analyses, nuclear proteins were resuspended in loading buffer, heated for 10 minutes at 95°C, and separated by SDS-PAGE (12% gels). Proteins were then transferred in Towbin buffer onto a PVDF membrane and probed with specific antibodies (Diagenode rabbit anti-H2A.Zac or Diagenode rabbit anti-H3K9ac [Diagenode, Liège, Belgium]). Secondary detection was performed using HRP-conjugated pAbs and enhanced chemoluminescence (ECL) substrate (Pierce/Thermo-Fisher, Rockford, Illinois, USA).

### Histone acetyltransferase/histone deacetylase activity assays

For histone acetyltransferase (HAT)/histone deacetylase (HDAC) activity assays, cells were counted by flow cytometry. HAT activity was assessed in MMH-D3 and HBV-Met nuclear extracts corresponding to 10.000 cells using the Epigentek EpiQuick HAT activity assay for ELISA (Epigentek, Farmingdale, NY, USA). Prior to absorbance detection at OD 450, HAT activity within those nuclear extracts was used to acetylate an immobilized histone substrate in the presence of acetyl Co-A. Then, acetylated substrates were recognized by means of specific antibodies and quantified by colorimetry. Histone deacetylase activities of either HDAC classes I/II or class III (sirtuins) were measured by means of luminescence reporter assays (Promega, Madison, WI, USA) using 10.000 cells for the class I/II HDAC assay (Promega HDAC Glo) or 20.000 cells for the sirtuin assay (Promega Sirt Glo). Prior to this, both MMH-D3 and HBV-Met cells were diluted in serum-free medium to a final volume of 100 μl per well. For HDAC and sirtuin assays, diluted cells, developer reagent and enzyme inhibitors were added and luminescence was detected at discrete time points using a Promega GloMax microplate reader (Promega, Madison, WI, USA).

### Chromatin accessibility assays

For *Micrococcus* nuclease assays, whole cell lysates were layered onto a 1.2 M sucrose cushion (1.2 M sucrose in 60 mM, KCl 15 mM NaCl, 5 mM MgCl_2_, 0.1 mM ethylene glycol tetraacetic acid (EGTA), 15 mM Tris–HCl (pH 7.5), 0.5 mM DTT, 0.1 mM phenylmethanesulfonylfluoride (PMSF), and 3.6 ng/mL aprotinin) and centrifuged as described elsewhere [41]. Nuclei were collected and homogenized in *Micrococcus* nuclease (MNase) digestion buffer (0.32 M sucrose, 50 mM Tris–HCl (pH 7.5), 4 mM MgCl_2_, 1 mM CaCl_2_, and 0.1 mM PMSF). MNase digest time courses of each 1 × 10^6^ cells were performed for 0, 1, 2, 4, 8, 16 or 20 minutes with 0.15 U/μL MNase (Thermo Fisher Scientific, Waltham, MA, USA). The reactions were stopped by directly mixing 50 μL phenol:chloroform:isoamylalcohol with each sample followed by DNA extraction. From the aqueous phase DNA was precipitated with isopropanol, resolved in water and DNA fragments were analyzed on an Agilent Bioanalyzer 2100 using a DNA chip.

### Chromatin immunoprecipitation

Chromatin isolation and chromatin immunoprecipitation (ChIP) analyses were carried out as described [42]. Antibodies used for ChIP were directed against H2A.Zac (Diagenode, Liège, Belgium), H3K4me1 (Diagenode, Liège, Belgium), H3K4me3 (Diagenode, Liège, Belgium), H3K9ac (Active Motif, Carlsbad, CA, USA), H3K9me3 (Active Motif, Carlsbad, CA, USA), H3K9me3S10ph (Diagenode, Liège, Belgium) or H3K27me3 (Active Motif, Carlsbad, CA, USA). Relative enrichment of precipitated sequences in ChIP was assessed using qPCR.

## Electronic supplementary material

Additional file 1: **Restitution of gene expression and histone acetylation signatures altered by hepatitis B virus through antiviral microRNA-like molecules in nontransformed murine hepatocytes.** (PDF 10 MB)

## Abbreviations

Ac: acetylation
ACTB: beta-actin
cccDNA: covalently closed circular DNA
CDH1: cadherin 1
CDKN1A: cyclin-dependent kinase inhibitor 1A
ChIP: chromatin immunoprecipitation
DNA: desoxyribonucleic acid
DNMT: DNA methyltransferase
ECL: enhanced chemoluminescence
EGTA: ethylene glycol tetraacetic acid
FHIT: fragile histidine triad protein
GAPDH: glyceraldehyde-3-phosphate dehydrogenase
GSTP1: glutathione S-transferase pi 1
GUSB: beta-glucuronidase
H2A: histone 2A
H3: histone 3
H4: histone 4
HAT: histone acetyltransferase
HBsAg: hepatitis B surface antigen
HBV: hepatitis B virus
HBV-Met: hepatitis B virus met murine hepatocytes
HBx: hepatitis B X protein
HCC: hepatocellular carcinoma
HDAC: histone deacetylase
HepG2: hepatocellular carcinoma G2 (cell line)
hsa: Homo sapiens
IGF2: insulin-like growth factor 2
K: lysine
MCL1: myeloid cell leukemia sequence 1
me: methylation
MeDIP: methylated DNA immunoprecipitation
miR: microRNA
MMH: met murine hepatocytes
MNase: *Micrococcus* nuclease
NAD^+^: nicotinamide adenine dinucleotide
ORF: open reading frame
ph: phosphorylation
PMSF: phenylmethanesulfonylfluoride
PTK2: protein tyrosine kinase 2
PTM: post-translational modification
qPCR: quantitative real-time polymerase chain reaction
RELN: reelin
RNA: ribonucleic acid
S: Serine
S/MAR: scaffold/matrix attached region
SF1: splicing factor 1
shRNA: short-hairpinRNA
SIRT1: silent mating type information regulation 2 homolog 1
TSA: trichostatin A
TSH2B: testis-specific histone 2B
3TC: 2',3'-dideoxy-3'-thiacytidine
5hmeC: 5-hydroxymethylcytosine
5meC: 5-methylcytosine.
