# Supplementary material for: Restitution of gene expression and histone acetylation signatures altered by hepatitis B virus through antiviral microRNA-like molecules in nontransformed murine hepatocytes
Source: Clin Epigenetics. 2014 Nov 14;6(1):26. doi: 10.1186/1868-7083-6-26 (PMC4391130; doi:10.1186/1868-7083-6-26)
Supplement: Supplementary file 1 — Additional file 1: Restitution of gene expression and histone acetylation signatures altered by hepatitis B virus through antiviral microRNA-like molecules in nontransformed murine hepatocytes. (PDF 10 MB) [file 13148_2014_90_MOESM1_ESM.pdf]

Additional file 1 - ‘Restitution of gene expression and histone acetylation signatures altered by hepatitis B virus through anti-viral miR-like molecules in non-transformed murine hepatocytes’

Andreas CW Jenke*,[1], Kai O Hensel*,[1], Andreas Klein[1], Lisa Willuhn[1], Susanna Prax[1], Patrick P Weil[1], Theodor Winkler[1], Timo Deba[1], Valerie Orth[1], Armin Baiker[2], Stefan Wirth[1], Jan Postberg†,[1]

[1]Department of Paediatrics, HELIOS Medical Centre Wuppertal, Centre for Clinical and Translational Research (CCTR), Witten/Herdecke University, Faculty of Health, Centre for Biomedical Education and Research (ZBAF), Wuppertal, Germany

[2]Bavarian Health and Food Safety Authority, Veterinaerstrasse 2, D-85764 Oberschleißheim, Germany

Figure S1

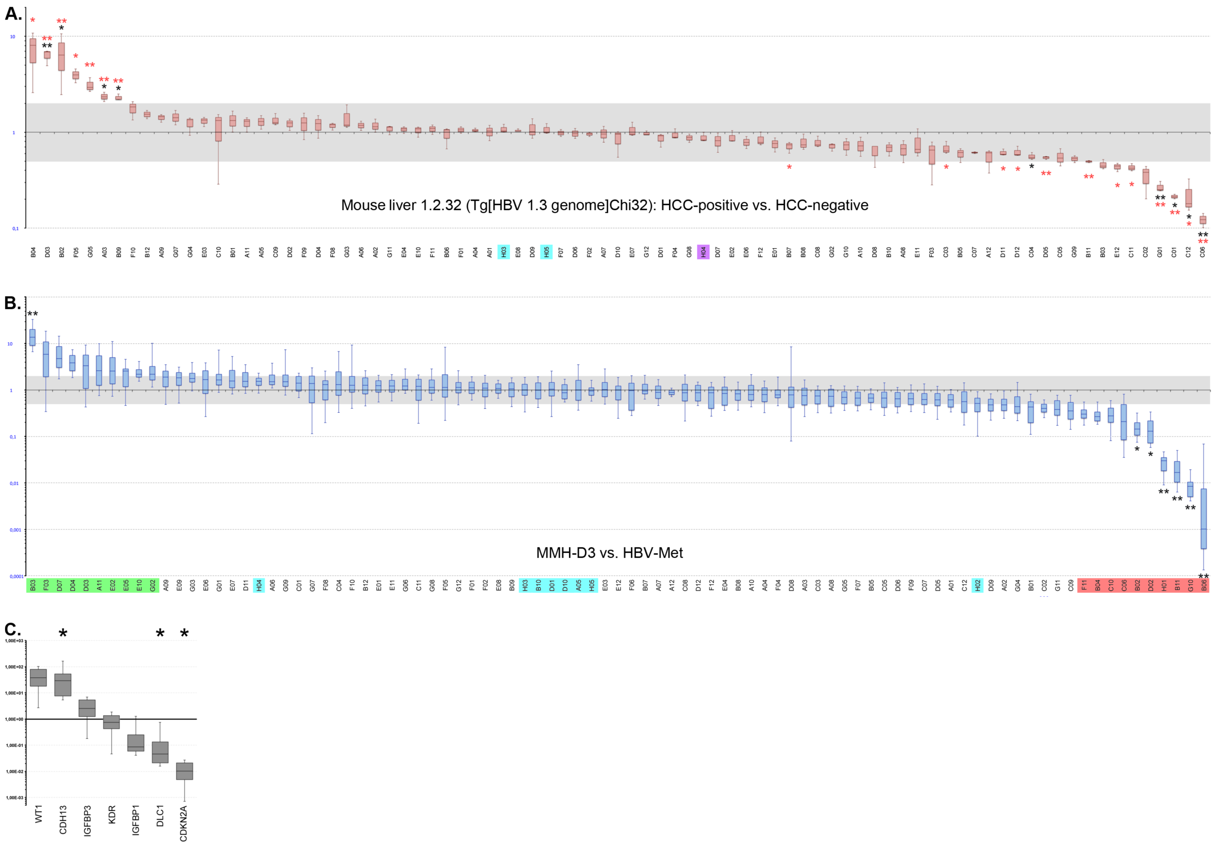

Comparison of differential mRNA accumulation between murine HBV-positive mice liver samples with or without HCC, mouse cell lines and humans.

A. Relative enrichment of mRNA in 1.2.32 (Tg[HBV 1.3 genome]Chi32) mice (HCC positive [n=5] versus HCC-negative [n=5]). *HSP90AB1*, *ACTB* and *GAPDH* were used for normalization (Cyan and purple shaded). **: p≤0.01; *: 0.01≤p≤0.05. Black asterisks: *HSP90AB1*, *ACTB* and *GAPDH* were used for normalization. Red asterisks: *HSP90AB1* and *ACTB* were used for normalization (Boxplots not shown). **B.** Relative enrichment of mRNA in MMH-D3 versus HBV-Met. H02 to H05 were used for normalization. **C.** Abundance of selected mRNAs in human liver from juvenile patients with chronic hepatitis B (non-HCC; n=3) versus HBV-negative specimens (n=3). Statistics and coloring correspond to Figure 1.

Figure S2

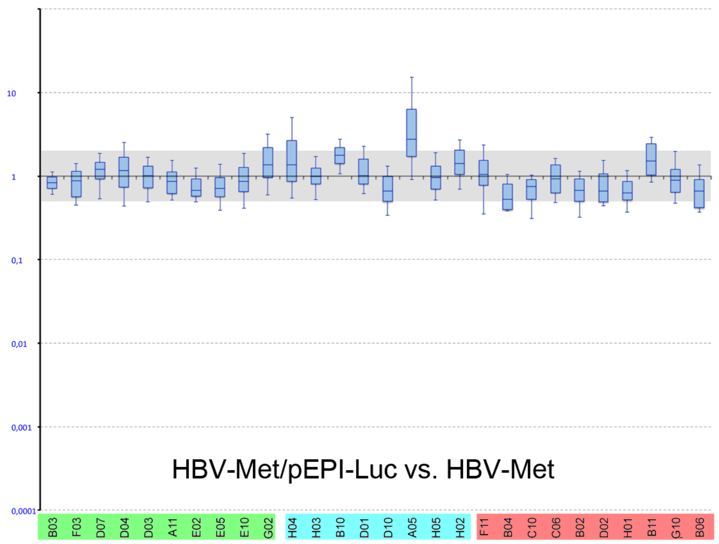

Gene expression analyses in HBV-Met transfected with the shRNA-cassette deficient pEPI-luciferase vector versus untreated HBV-Met. Statistics and coloring correspond to Figure 1.

Figure S3

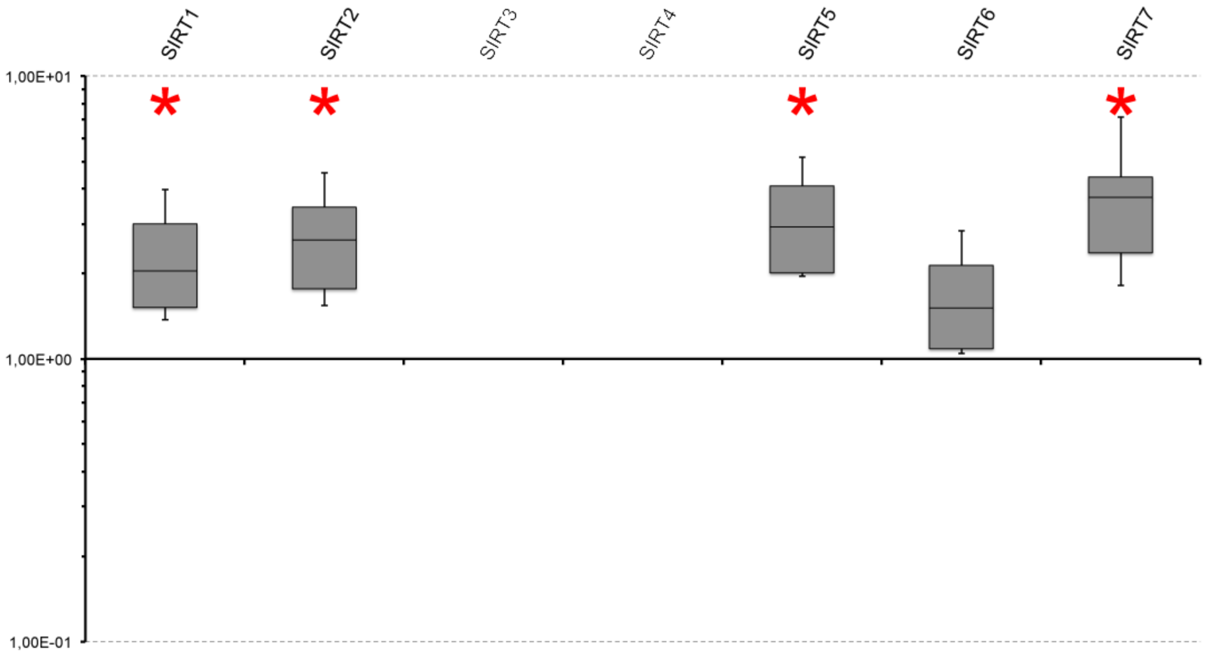

Accumulation of SIRT1 -7mRNAs in HBV-Met cells compared with MMH-D3 cells. The mRNAs of *SIRT1*, *SIRT2*, *SIRT4* and *SIRT7* were slightly but significantly (*: $0.01 \leq p \leq 0.05$) elevated in HBV-Met cells when compared with MMH-D3 cells, whereas *SIRT3* and *SIRT4* could not be detected in both cell lines. Statistics correspond to Figure 1.

Figure S4

Position	Unigene	Refseq	Symbol	Description
A01	Mm.27681	NM_009615	Adam17	A disintegrin and metallopeptidase domain 17
A02	Mm.6645	NM_009652	Akt1	Thymoma viral proto-oncogene 1
A03	Mm.439874	NM_007426	Angpt2	Angiopoietin 2
A04	Mm.19904	NM_007527	Bax	Bcl2-associated X protein
A05	Mm.257460	NM_009741	Bcl2	B-cell leukemia/lymphoma 2
A06	Mm.238213	NM_009743	Bcl2l1	Bcl2-like 1
A07	Mm.235081	NM_007544	Bid	BH3 interacting domain death agonist
A08	Mm.335659	NM_007465	Birc2	Baculoviral IAP repeat-containing 2
A09	Mm.8552	NM_009689	Birc5	Baculoviral IAP repeat-containing 5
A10	Mm.336851	NM_009812	Casp8	Caspase 8
A11	Mm.284248	NM_013653	Ccl5	Chemokine (C-C motif) ligand 5
A12	Mm.273049	NM_007631	Ccnd1	Cyclin D1
B01	Mm.333406	NM_009829	Ccnd2	Cyclin D2
B02	Mm.35605	NM_009864	Cdh1	Cadherin 1
B03	Mm.334841	NM_019707	Cdh13	Cadherin 13
B04	Mm.195663	NM_007669	Cdkn1a	Cyclin-dependent kinase inhibitor 1A (P21)
B05	Mm.2958	NM_009875	Cdkn1b	Cyclin-dependent kinase inhibitor 1B

B06	Mm.4733	NM_009877	Cdkn2a	Cyclin-dependent kinase inhibitor 2A
B07	Mm.336848	NM_009805	Cflar	CASP8 and FADD-like apoptosis regulator
B08	Mm.291928	NM_007614	Ctnnb1	Catenin (cadherin associated protein), beta 1
B09	Mm.1401	NM_009911	Cxcr4	Chemokine (C-X-C motif) receptor 4
B10	Mm.29629	NM_001001602	Dab2ip	Disabled homolog 2 (Drosophila) interacting protein
B11	Mm.210875	NM_015802	Dlc1	Deleted in liver cancer 1
B12	Mm.18036	NM_007891	E2f1	E2F transcription factor 1
C01	Mm.252481	NM_010113	Egf	Epidermal growth factor
C02	Mm.8534	NM_007912	Egfr	Epidermal growth factor receptor
C03	Mm.258397	NM_177821	Ep300	E1A binding protein p300
C04	Mm.5126	NM_010175	Fadd	Fas (TNFRSF6)-associated via death domain
C05	Mm.1626	NM_007987	Fas	Fas (TNF receptor superfamily member 6)
C06	Mm.397619	NM_010210	Fhit	Fragile histidine triad gene
C07	Mm.389712	NM_010228	Flt1	FMS-like tyrosine kinase 1
C08	Mm.297906	NM_008057	Fzd7	Frizzled homolog 7 (Drosophila)
C09	Mm.1360	NM_008655	Gadd45b	Growth arrest and DNA-damage-inducible 45 beta
C10	Mm.299292	NM_013541	Gstp1	Glutathione S-transferase, pi 1
C11	Mm.267078	NM_010427	Hgf	Hepatocyte growth factor
C12	Mm.254493	NM_020259	Hhip	Hedgehog-interacting protein
D01	Mm.334313	NM_008284	Hras1	Harvey rat sarcoma virus oncogene 1
D02	Mm.3862	NM_010514	Igf2	Insulin-like growth factor 2
D03	Mm.21300	NM_008341	Igfbp1	Insulin-like growth factor binding protein 1
D04	Mm.29254	NM_008343	Igfbp3	Insulin-like growth factor binding protein 3
D05	Mm.4952	NM_010570	Irs1	Insulin receptor substrate 1
D06	Mm.263396	NM_010578	Itgb1	Integrin beta 1 (fibronectin receptor beta)
D07	Mm.285	NM_010612	Kdr	Kinase insert domain protein receptor
D08	Mm.255219	NM_010703	Lef1	Lymphoid enhancer binding factor 1
D09	Mm.1639	NM_008562	Mcl1	Myeloid cell leukemia sequence 1
D10	Mm.86844	NM_008591	Met	Met proto-oncogene
D11	Mm.4619	NM_008628	Msh2	MutS homolog 2 (E. coli)
D12	Mm.343101	NM_010829	Msh3	MutS homolog 3 (E. coli)
E01	Mm.130883	NM_026002	Mtdh	Metadherin
E02	Mm.2444	NM_010849	Myc	Myelocytomatosis oncogene
E03	Mm.256765	NM_008689	Nfkb1	Nuclear factor of kappa light polypeptide gene enhancer in B-cells 1, p105
E04	Mm.400954	NM_010937	Nras	Neuroblastoma ras oncogene
E05	Mm.379474	NM_177906	Opcml	Opioid binding protein/cell adhesion molecule-like
E06	Mm.221403	NM_011058	Pdgfra	Platelet derived growth factor receptor, alpha polypeptide
E07	Mm.7906	NM_023371	Pin1	Protein (peptidyl-prolyl cis/trans isomerase) NIMA-interacting 1
E08	Mm.245395	NM_008960	Pten	Phosphatase and tensin homolog
E09	Mm.292547	NM_011198	Ptgs2	Prostaglandin-endoperoxide synthase 2
E10	Mm.254494	NM_007982	Ptk2	PTK2 protein tyrosine kinase 2
E11	Mm.24163	NM_023258	Pycard	PYD and CARD domain containing
E12	Mm.292510	NM_009007	Rac1	RAS-related C3 botulinum substrate 1
F01	Mm.12091	NM_019713	Rassf1	Ras association (RalGDS/AF-6) domain family member 1
F02	Mm.273862	NM_009029	Rb1	Retinoblastoma 1
F03	Mm.425236	NM_011261	Reln	Reelin
F04	Mm.757	NM_016802	Rhoa	Ras homolog gene family, member A

F05	Mm.378894	NM_019732	Runx3	Runt related transcription factor 3
F06	Mm.19155	NM_009144	Sfrp2	Secreted frizzled-related protein 2
F07	Mm.100399	NM_008540	Smad4	MAD homolog 4 (Drosophila)
F08	Mm.34407	NM_001042660	Smad7	MAD homolog 7 (Drosophila)
F09	Mm.130	NM_009896	Socs1	Suppressor of cytokine signaling 1
F10	Mm.3468	NM_007707	Socs3	Suppressor of cytokine signaling 3
F11	Mm.249934	NM_011486	Stat3	Signal transducer and activator of transcription 3
F12	Mm.4269	NM_013685	Tcf4	Transcription factor 4
G01	Mm.10109	NM_009354	Tert	Telomerase reverse transcriptase
G02	Mm.137222	NM_031199	Tgfa	Transforming growth factor alpha
G03	Mm.248380	NM_011577	Tgfb1	Transforming growth factor, beta 1
G04	Mm.172346	NM_009371	Tgfbr2	Transforming growth factor, beta receptor II
G05	Mm.38049	NM_021297	Tlr4	Toll-like receptor 4
G06	Mm.193430	NM_020275	Tnfrsf10b	Tumor necrosis factor receptor superfamily, member 10b
G07	Mm.1062	NM_009425	Tnfsf10	Tumor necrosis factor (ligand) superfamily, member 10
G08	Mm.222	NM_011640	Trp53	Transformation related protein 53
G09	Mm.282184	NM_009505	Vegfa	Vascular endothelial growth factor A
G10	Mm.389339	NM_144783	Wt1	Wilms tumor 1 homolog
G11	Mm.259879	NM_009688	Xiap	X-linked inhibitor of apoptosis
G12	Mm.221992	NM_009534	Yap1	Yes-associated protein 1
H01	Mm.3317	NM_010368	Gusb	Glucuronidase, beta
H02	Mm.299381	NM_013556	Hprt	Hypoxanthine guanine phosphoribosyl transferase
H03	Mm.2180	NM_008302	Hsp90ab1	Heat shock protein 90 alpha (cytosolic), class B member 1
H04	Mm.343110	NM_008084	Gapdh	Glyceraldehyde-3-phosphate dehydrogenase
H05	Mm.328431	NM_007393	Actb	Actin, beta

Genes in focus and numbering (column 1) assigned to Figures 1-3 and Additional file 1: Figures S1-S2.
